# An Innovative Feedback Tool Leading to Improved Faculty Feedback and Positive Reception by Residents

**DOI:** 10.5811/westjem.2019.10.44302

**Published:** 2019-12-19

**Authors:** Raquel Harrison, Alina Tsyrulnik, David Brian Wood, Ryan F. Coughlin, David Della-Giustina, Katja Goldflam

**Affiliations:** * Bridgeport Hospital, Department of Emergency Medicine, Bridgeport, Connecticut; † Yale University School of Medicine, Department of Emergency Medicine, New Haven, Connecticut; ‡ St. Joseph Medical Center, Department of Emergency Medicine, Stockton, California

## Abstract

**Introduction:**

In 2012 the Accreditation Council for Graduate Medical Education implemented trainee milestones as tools for clinical competency committees to use for evaluation, feedback, remediation, and promotion purposes. Prior to this innovation, there has not been an adequate method to capture, organize, and graphically illustrate the evaluations by attendings in a simple, fast and organized fashion.

**Methods:**

We created a novel, web-based, mobile-friendly evaluation tool to help fill this identified gap. The survey-based program creates a milestone-based evaluation, takes only a few minutes to complete, and easily collates the results in a graphic format creating an individualized “dashboard.” The dashboard is then used by both trainees and their evaluators as a feedback platform.

**Results:**

With the implementation of the dashboard, educational leadership has noted an increase in the number of submitted evaluations of residents and the amount of face-to-face feedback given by attendings to residents. A post-implementation survey of the residents revealed that they found the dashboard-provided feedback more helpful than prior modes of feedback, although the number of evaluations was still too few.

**Conclusion:**

The use of our feedback dashboard is useful to multiple targeted end-users, including general faculty evaluators, program leadership, and the residents themselves for gathering formative feedback that is specific and timely. This tool is adaptable and likely generalizable to other residency programs and specialties.

## INTRODUCTION

The Accreditation Council on Graduate Medical Education (ACGME) requires residency leadership to evaluate residents and inform the residency clinical competency committee (CCC), in order for them to evaluate each resident using the ACGME milestone framework for assessment (essential competencies defined by each specialty). Additionally, the CCC should seek to provide formative feedback to residents for goal-directed self-improvement and to encourage reflective conversations.^1^ Feedback should be ongoing, dynamic, encourage self-reflection, and provide a structure for desired performance.^2^

Emergency medicine (EM) is a unique clinical environment. The rapid pace of clinical care provides the resident with a rich *in vivo* context for observational learning from undifferentiated patients, procedures, resuscitations, and challenging patient-care team interactions. Unfortunately, there is a lack of satisfaction across EM residents regarding quantity and quality of real-time feedback.^3^ Previous data suggest that visual aid modalities may help reinforce delivery of constructive feedback.^4^ To our knowledge, dashboards have been described as opportunities to visualize data for CCCs and semi-annual performance evaluations but not to increase transparency of information to learners on an ongoing basis.^5^ This study group sought an innovative way to improve assessment satisfaction and quality, verbal feedback, and to understand resident performance on both individual and group levels using a survey-driven dashboard.

## OBJECTIVES

The objectives of this educational innovation were threefold. The first was to develop a resident evaluation tool that would allow ease of use by the attending physicians. This tool would increase attending participation in feedback and encourage the timeliness of feedback as the evaluation was completed, preferably at the end of the associated clinical shift. Second, we sought to develop a feedback tool that was thorough enough to evaluate the resident using the ACGME Milestone framework. Finally, we aimed to develop a dashboard that could summarize and display the feedback in a clear and easy-to-read format that would be available to the resident in real time.

## CURRICULAR DESIGN

The study presented was performed at a four-year, academic EM residency program with 15 residents per class. Although the residents rotate at two sites, a university and a community hospital (each with its own faculty), the study was done with only the university faculty (52 total faculty) evaluating residents on their performance at the main site (university hospital). The description of this feedback and assessment innovation received exemption from the institutional review board.

Approximately three years ago, a need for improved compliance with evaluation of resident clinical performance was identified within the department (Table 1). Two focus group sessions were conducted: one with faculty, to communicate possible reasons for poor compliance; and one with residents, to understand their perceptions of the quality of evaluations received. The faculty focus group included nine faculty members from different sections within the department (department of EM sections include: administration, education, emergency medical services, global health, research, and ultrasound). The resident focus group included nine members from the rising postgraduate year (PGY) 2 and rising PGY 3 residency classes.

After analyzing concerns discussed during these focus groups, two internal surveys were created to further understand the problem within the department on a broader scale and sent to the faculty and residents (Appendix A and B respectively). The response rate for the faculty survey was 38 out of 52 (73%), and that for the residents was 33 out of 60 (55%). As these surveys were created de novo, based on focus groups, there was no validation process for them. From the survey results, an evaluation tool was developed that faculty could access via a mobile device. This tool used a survey system available within our university, Qualtrics, a licensed survey design system that allows online survey design and randomization of survey questions.

A simplified “beta” version of the tool was initially tested by the residency leadership team for approximately two months. Minor changes were made, and it was then distributed to the faculty as a whole (referred to as “Version 1” in Table). After sending an access link via e-mail, faculty were introduced to the tool during a faculty meeting and encouraged to “add a shortcut to the homescreen” on their mobile device for ease of mobile access. Less than 10 minutes of training was required to familiarize users with the tool. Additionally, a QR (quick response) matrix barcode was posted in the faculty workroom to visually remind them of the feedback tool and to allow them an additional method of easy access to the tool. The residents were surveyed after six months to assess satisfaction with the new evaluation system (Appendix C). The response rate to the survey was 43% (26 out of 60 residents).

Because “Version 1” of the survey tool provided proof of concept, modifications were then made to the survey to capture more information. Currently, the Qualtrics-based evaluation tool is a web-based survey form easily accessed on a mobile device or computer, taking only a few minutes to complete. This process is initiated by attending physicians, but residents are also encouraged to prompt attendings to use the tool.

**Figure 1:**
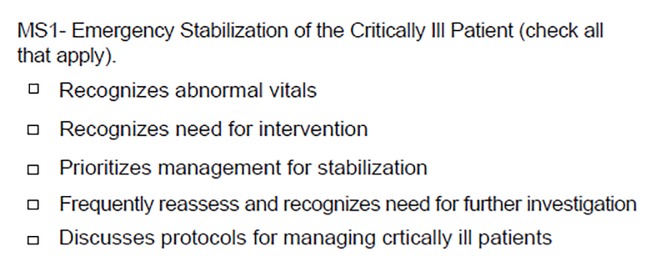
Sample question from survey tool based on emergency medicine milestone 1.

In an effort to maximize the clinical applicability of the 23 EM milestones, they were subdivided into procedural and non-procedural subcategories. Further, within each milestone the verbiage of the individual levels was truncated to extract only the clinically relevant aspects of the competencies (Figure 1). Language was reviewed and created via consensus effort discussion among the residency leadership group.

When using the platform, the evaluator is given two randomized questions from a pool of 17 questions that represent the non-procedural milestones. The evaluator is not forced to place the resident on a scale, but rather check all competencies within each milestone that were met. The evaluator is then prompted with a list of possible milestone procedures that may have been supervised. If observed, they can evaluate the procedure performed based on the milestone competencies. More than one procedure can be evaluated during a single evaluation. The evaluation finishes with two qualitative questions that can be dictated or typed if using a mobile device: “1. strengths of shift (cite example);” and “2. items to work on/medical topic to focus on (cite example).” A prompt is then provided to assess whether verbal feedback was provided to the resident and whether positive, constructive, or both were discussed.

**Figure 2:**
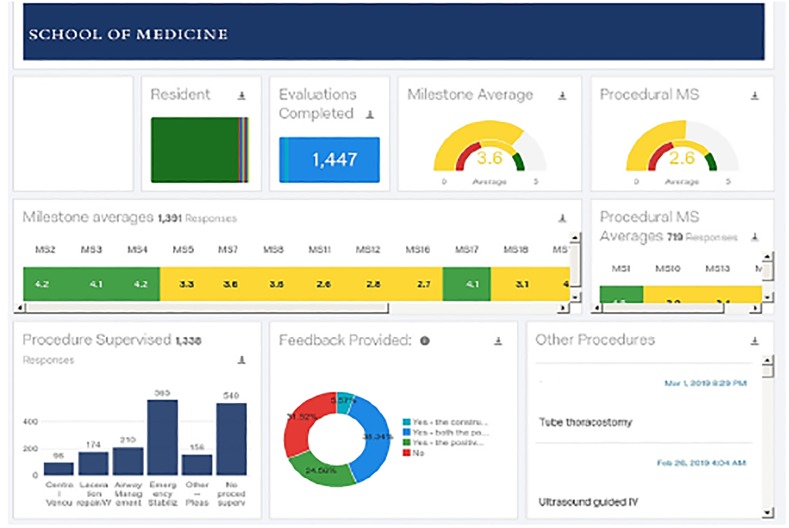
Sample of dashboard screenshot.

The data collected from these evaluations were mapped in real-time into a visually simplified dashboard (part of Qualtrics Vocalize functionality) that allows residents and residency leadership to identify trends of success and deficiency, and to access specific qualitative feedback (Figure 2).

Figure 2 shows a visual representation of compilation of all feedback given for an individual or a group of residents over a time period that can be selected. An additional feature of the dashboard is the ability to include evaluation data from other sources, such as evaluations of residents by nurses. Those sources can use a different set of evaluation questions, as they do at our institution, as long as they use the Qualtrics survey tool. Access to the survey is granted via an e-mailed link to nurses. The use of this software was donated for the pilot of this project by Qualtrics.

## IMPACT/EFFECTIVENESS

**Figure 3:**
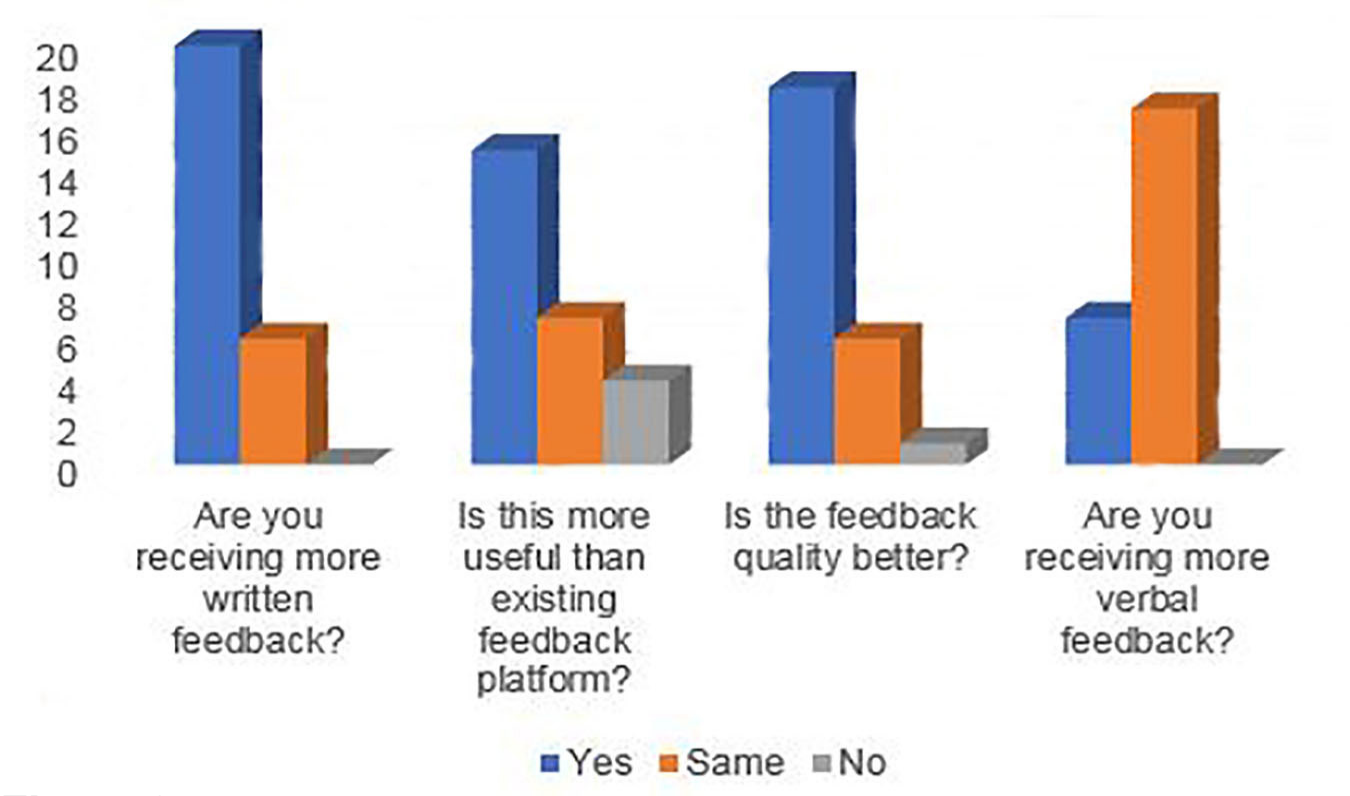
Resident survey results. Graphical representation of responses by residents to a survey assessing their satisfaction with the dashboard six months after its use was initiated (Appendix C).

Compliance with evaluations, as measured by the number of faculty filling out at least one evaluation, increased from approximately 18 out of 52 (34.6%) before “Version 1” implementation to 45 out of 56 (86.5%) thereafter (p<0.001). Additionally, 26 out of 60 residents (43%) responded to a survey six months after the dashboard was in use to assess satisfaction with this tool and reported finding feedback via the dashboard more specific and more “useful” in comparison to the other evaluation system (Medhub) used at our institution. They cited receiving more written feedback with the new system, although the majority felt like the amount of verbal feedback remained static (Figure 3 and Appendix C).

**Figure 4:**
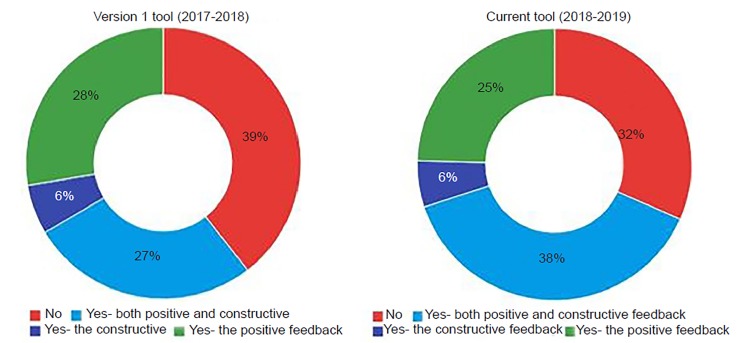
Did you have a face-to-face discussion regarding this feedback with the resident? Demonstrated decrease in “No feedback given” and increase in “Constructive feedback given.”

Between “Version 1” implementation and the current version, attending physicians have self-identified an increase in the amount of verbal feedback that is being communicated. Analysis of the prompt: *“Did you have a face-to-face discussion regarding this feedback with the resident?****”*** revealed an improvement in the answer “No” from 39.4% (n= 494) during the “Version 1” phase to 31.52% (n = 1364) in the current model (p = 0.001). The amount of “constructive” feedback also increased from 32.92% (n = 494) to 43.91% (n = 1364) (p<0.001) (Figure 4).

There is a general sense that the dashboard was well received among the residency leadership and the CCC members, as it allowed them to review residents during meetings and easily compare them to their peer group in order to assure proper progress through residency. It is particularly useful in CCC meetings (projected on the conference room screen), and during individual meetings with residents. Based on the small response rate to the post-deployment resident survey (43%), the residents reported that they interacted with the dashboard less frequently than we anticipated. Residents who routinely read their evaluations reported that the information was more helpful in real time, but still thought that the overall number of individual evaluations by faculty were too few.

In summary, this Qualtrics-based survey tool has improved faculty engagement in evaluation and feedback while providing more specific information to the residents. Compared to prior dashboards described in publication,^5^ the tool presented here is a survey-based program that creates a milestone-based evaluation (including the ability to select procedures observed), takes only a few minutes to complete, and easily collates the results in a graphic format creating an individualized “dashboard.” In addition, its application is not only for use by the CCC, but as feedback to residents on their performance. Because the tool is based on a survey system, a similar tool could likely be implemented within other residencies (without need for embedded coding knowledge) that are striving to engage more faculty; it would also be relevant to other procedural specialties that are observation-rich and where more frequent, smaller evaluations better inform the whole understanding of resident performance.

Through this new platform, and an effort to evaluate its effects, there is preliminary evidence suggesting improvement in the culture of feedback in our department by making a few key changes. We created a tool that can be accessed on a mobile device, is simple to use, and in which data collection is brief, asking the evaluator to complete what they are able to within the structure of their time constraints. This allows accumulation of data over time, even if small in amount, which was an improvement at this program. By taking advantage of randomization and the ability to select any procedure that was observed, we have been able to collect the breadth of information required to properly assess milestones despite each individual evaluation remaining brief. In addition, the dashboard displays the information in an easily understandable format and allows the residency leadership and CCC to identify trends for individuals and groups of residents quickly. Lastly, by providing a prompt toward feedback at the beginning, and by simply asking at the end of the form whether the faculty members have engaged in face-to-face conversations with their residents, the number of conversations over time (as self-reported by the evaluators themselves) has significantly increased.

## LIMITATIONS

The authors acknowledge several limitations to this study. First, there are no baseline formal measurements of written and face-to-face evaluations and their frequency before the intervention. There is potentially a pre-existing upward trend in evaluation numbers in response to resident feedback. The assumption made by this group is that the frequency and rate of increase in feedback was unacceptably low. The measured improvement in feedback frequency could be due to a larger focus on this problem area or the discussions on how to use the new tool, rather than the implemented changes themselves. The evaluation platform and dashboard were implemented at a single center and used only by faculty at the university residency site. Furthermore, the surveys used by the team before and after implementation were not validated and had relatively low response rates. The post-implementation data collected focused on the residents’ perception of the feedback they received after its used, but not on the raw numbers of evaluations per resident per specific time period.

## CONCLUSION

While we can report a trend of improved feedback frequency and quality, improving the culture of feedback requires significant evidence of sustained and well-integrated change. This is a direction for future study as well as an effort to evaluate similar tools at other institutions and across specialties.

## Supplementary Information



## Figures and Tables

**Table 1:** Timeline of survey tool development.

June 2016	Focus groups: faculty and residents
July 2016	Departmental surveys/needs assessment of faculty and residents
July 2016	Test version using residency leadership only
September 2016	Version 1 launched
September 2017	Current evaluation tool
September 2018	Dashboard launched
