# Supplementary material for: An Innovative Feedback Tool Leading to Improved Faculty Feedback and Positive Reception by Residents
Source: West J Emerg Med. 2019 Dec 19;21(1):47–51. doi: 10.5811/westjem.2019.10.44302 (PMC6948690; doi:10.5811/westjem.2019.10.44302)
Supplement: Supplementary file 1 [file wjem-21-47-s001.docx]

| Appendix A: Faculty Survey based on Focus Group | |
| --- | --- |
| Please take approximately 10 minutes to complete this survey.  We are seeking to improve the feedback process of residents by attending physicians within the Department of Emergency Medicine.  Our goals: -Encourage more formative and summative feedback of the residents by attending physicians. -Increase conversation and real-time feedback between residents and attending physicians. -Increase compliance with required national feedback delivery standards for formal evaluation.  -More timely identification of strengths and deficiencies in resident performance. | |
| Q1 | My current compliance with Resident evaluation is:   \|  \| Never \|  \|  \|  \| Always \| \| --- \| --- \| --- \| --- \| --- \| --- \|  \|  \| 0 \| 10 \| 20 \| 30 \| 40 \| 50 \| 60 \| 70 \| 80 \| 90 \| 100 \| \| --- \| --- \| --- \| --- \| --- \| --- \| --- \| --- \| --- \| --- \| --- \| --- \|  \| Compliance () \| 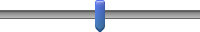 \| \| --- \| --- \| |
| Q2 |  |
| Q3 | The biggest obstacles to my completion of Resident evaluations are (choose all that apply):  The web-based platform (ie. Medhub, e-value, etc.)  Too many questions  Too many evaluations pending  Too general  Not specific to who I have worked with  Questions are too long  Too many clicks  Lack of benefit for resident  Lack of interest for me  Lack of positive incentive for me  Lack of negative incentive for me  Other: (10) |
| Q4 | When I fill out evaluations, I prefer and find more value in:  Writing narrative descriptions of performance  Using scales and ranking skills of different competencies/ milestones  Combination of the above - using both narratives and scales  Other: (4) ________________________________________________ |
| Q5 | When I give verbal feedback, I prefer and find more value in:  Approaching the Resident and giving my thoughts  Having the Resident ask me and prompt me for feedback  Other: (3) ________________________________________________ |
| Q6 | How often do you give verbal feedback while on shift with the Resident?   \|  \| Never \|  \|  \|  \| Always \| \| --- \| --- \| --- \| --- \| --- \| --- \|  \|  \| 0 \| 10 \| 20 \| 30 \| 40 \| 50 \| 60 \| 70 \| 80 \| 90 \| 100 \| \| --- \| --- \| --- \| --- \| --- \| --- \| --- \| --- \| --- \| --- \| --- \| --- \|  \| POSITIVE - What Resident did well () \| 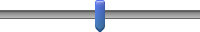 \| \| --- \| --- \| \| CONSTRUCTIVE -What Resident did not do well () \| 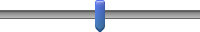 \| |
| Q7 | The biggest obstacles to giving VERBAL feedback are (choose all that apply):  Not enough time or too busy  Constructive feedback is hard to give  Constructive feedback is poorly received  Fear of damaging relationship with Resident  Positive feedback is too general  I do not like confrontation  I do not think the Resident is interested  At times, I do not feel like I have sufficient tools to give effective feedback  Not enough contact time with the Resident  Other: |
| Q8 | I have been supervising Residents in the Emergency Department for:  Less than 1 year  Less than 2 years  Less than 5 years  5-10 years  Greater than 10 years |
| Q9 | I carry a hospital issued smart phone or personal smart phone while working clinically on shift:  Yes - Personal Smartphone  Yes - Hospital issued Smartphone  Yes - Both  Neither |
| Q10 | I specifically use and am comfortable with:  iPhone/Apple  Android  Both  Neither |
| Q11 | If I had access to a mobile-app to evaluate the Residents I work with, I would be more likely to fill out evaluations:  Yes  No |
| Q12 | If I had access to a mobile-app to evaluate the Residents I work with, I would be more likely to have face-to-face conversations surrounding feedback:  Yes  No |
| Q13 | What would be the most successful way to notify you or prompt you to fill out an evaluation? (choose all that apply)  Resident face-to-face reminder  Postings in the office attending areas  Postings in attending areas of the Emergency Department  Push notifications from the Mobile-App itself  Automatic time-sensitive reminders  A reminder linked with the timing of your shift  Other: |
| Q14 | How many questions would you be willing to answer about each resident via a mobile application?  3  4  5  6  Other: |
| Q15 | How much time would you be willing to spend filling out a mobile-based evaluation form during/after a shift in the Emergency Department?  < 1 minute  1-2 minutes  < 3 minutes  < 5 minutes |
| Q16 | Would you like the option to verify and evaluate procedures on the same form?  Yes  No  Indifferent |
| Q17 | If you have access to a mobile-app, would you still use the current web-based application for more thorough evaluations and procedure verification?  Yes  Maybe  No |
| Q18 | What would make you most likely to use the mobile-app on a consistent basis? |
| Q19 | What type of questions would you like to see in the mobile-app evaluation? |
| Q20 | We are specifically looking for ideas to increase the ease of use of a mobile app for evaluation and feedback.  Please let us know any other thoughts or ideas. |

| Appendix B: Resident Survey based on Focus Group | |
| --- | --- |
| Please take approximately 10 minutes to complete this survey.  We are seeking to improve the feedback process between residents and attending physicians within the Department of Emergency Medicine.  Our goals: -Encourage more formative and summative feedback of the residents by attending physicians. -Increase conversation and real-time feedback between residents and attending physicians. -Increase compliance with required national feedback delivery standards for formal evaluation.  -More timely identification of strengths and deficiencies in resident performance. | |
| Q1 | My current level of training is:  EM-1  EM-2  EM-3  EM-4 |
| Q2 | My current compliance with attending evaluations is:   \|  \| Never \|  \|  \|  \| Always \| \| --- \| --- \| --- \| --- \| --- \| --- \|  \|  \| 0 \| 10 \| 20 \| 30 \| 40 \| 50 \| 60 \| 70 \| 80 \| 90 \| 100 \| \| --- \| --- \| --- \| --- \| --- \| --- \| --- \| --- \| --- \| --- \| --- \| --- \|  \| Compliance () \| 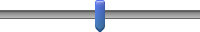 \| \| --- \| --- \| |
| Q3 | The biggest obstacles to **receiving WRITTEN**evaluations from attendings are (choose all that apply):  Inadequate amount of feedback to establish trends for improvement  Delayed feedback  Inadequate contact with different attendings  Increased feedback for outliers in performance  Other (please specify): |
| Q4 | The biggest obstacles to **giving WRITTEN** evaluations to attendings are (choose all that apply):  The web-based platform (ie. Medhub, e-value, etc.)  Too many questions  Too many evaluations pending  Too general  Not specific to who I have worked with  Questions are too long  Too many clicks  Lack of benefit for attending  Lack of interest for me  Lack of positive incentive for me  Lack of negative incentive for me  Other (please specify): |
| Q5 | When I fill out evaluations, I prefer and find more value in:  Writing narrative descriptions of performance  Using scales for performance - comparing peers  Combination of the above - using both narratives and scales  Other (please specify): |
| Q6 | How often do you receive **VERBAL** feedback?   \|  \| Never \|  \|  \|  \| Always \| \| --- \| --- \| --- \| --- \| --- \| --- \|  \|  \| 0 \| 10 \| 20 \| 30 \| 40 \| 50 \| 60 \| 70 \| 80 \| 90 \| 100 \| \| --- \| --- \| --- \| --- \| --- \| --- \| --- \| --- \| --- \| --- \| --- \| --- \|  \| POSITIVE - What you did well () \| 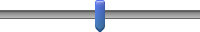 \| \| --- \| --- \| \| CONSTRUCTIVE -What you did not do well () \| 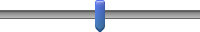 \| |
| Q7 | When I receive **VERBAL** feedback, I prefer and find more value in (check all that apply):  Attendings offering feedback without solicitation  Actively requesting feedback  Specific goals to work on before a shift  Detailed feedback  Other (please specify): |
| Q8 | The biggest obstacles to **receiving VERBAL**feedback are (choose all that apply):  Not enough time or too busy  Positive feedback is too general  I do not like confrontation  I do not like negative feedback  Not enough contact time with the attending  Other (please specify): |
| Q9 | I carry a hospital issued smart phone or personal smart phone while working clinically on shift:  Yes - Personal Smartphone  Yes - Hospital issued Smartphone  Yes - Both  Neither |
| Q10 | I specifically use and am comfortable with:  iPhone/Apple  Android  Both  Neither |
| Q11 | If I had access to a mobile-app to evaluate the attendings I work with, I would be more likely to fill out evaluations:  Yes  No |
| Q12 | What would be the most successful way to notify you or prompt you to fill out an evaluation? (choose all that apply)  Face-to-face reminder  Postings in the resident room or common areas  Push notifications from the Mobile-App itself  Automatic time-sensitive reminders  A reminder linked with the timing of your shift  Other (please specify): |
| Q13 | How many questions would you be willing to answer about each attending via a mobile application?  3  4  5  6  Other (please specify): |
| Q14 | How much time would you be willing to spend filling out a mobile-based evaluation form during/after a shift in the Emergency Department?  < 1 minute  1-2 minutes  < 3 minutes  < 5 minutes |
| Q15 | If you have access to a mobile-app, would you still use the current web-based application for more thorough evaluations and procedure verification?  Yes  Maybe  No |
| Q16 | Would you like to have procedure logging in the same app?  Yes  No  Indifferent |
| Q17 | What would make you most likely to use the mobile-app on a consistent basis? |
| Q18 | What type of questions would you like to see in the mobile-app evaluation? |
| Q19 | We are specifically looking for ideas to increase the ease of use of a mobile app for evaluation and feedback.  Please let us know any other thoughts or ideas_______________ |

| Appendix C: Resident Survey after “Version 1” of Survey Tool and Dashboard | |
| --- | --- |
| Please take approximately 3 minutes to complete this survey. You are now receiving feedback from a new evaluation process compared to MedHub. We would like to understand the changes. | |
| Q1 | How many evaluations did you receive?  0  1-2  3-4  5 or more |
| Q2 | In this report, do you feel like you are receiving more written feedback?  Yes, More feedback  No, Same amount  No, Less feedback |
| Q3 | Is this feedback more useful than MedHub?  Yes  Maybe  No |
| Q4 | Why? |
| Q5 | How is the quality of feedback when compared to MedHub?  Better  Same  Worse |
| Q6 | In the past 3 months, do you feel like you are receiving more verbal feedback?  Yes, More feedback  No, Same amount  No, Less feedback |
| Q7 | We welcome any ideas for improvement |
